# HoBi-Like Pestivirus and Reproductive Disorders

**DOI:** 10.3389/fvets.2020.622447

**Published:** 2020-12-22

**Authors:** Nicola Decaro

**Affiliations:** Department of Veterinary Medicine, University of Bari Aldo Moro, Valenzano, Italy

**Keywords:** HoBi-like pestivirus, cattle, reproduction, natural infections, experimental Infections

## Abstract

HoBi-like pestivirus (HoBiPeV) is an emerging group of pestiviruses that has been detected in cattle and other ruminants in South America, Europe, and Asia. Analogous to other bovine pestiviruses, namely bovine viral diarrhea (BVDV) 1 and 2, HoBiPeV is able to cause a variety of clinical forms that range from asymptomatic infections to fatal disease, having a great impact on cattle productions and causing substantial economic losses, mainly as a consequence of the occurrence of reproductive failures. The manuscript aims to provide an updated review of the currently available literature about the impact of HoBiPeV infection on cattle reproduction. The reproductive disorders observed in cattle due to natural and experimental infections caused by this virus are reported along with the few available *in-vitro* studies involving the reproductive tract. HoBiPeV should be considered among the bovine pathogens that impact on reproduction, but there is a need for more specific and sensitive diagnostic methods, while the cross-protection elicited by commercially available BVDV vaccines should be better investigated.

## Introduction

Pestivirus infections in cattle are associated with various clinical forms that include subclinical infections, immunosuppression, respiratory signs, gastroenteritis, reproductive failures, haemorrhagic and systemic disease, such as mucosal disease in persistently infected (PI) animals (1–3). Pestiviruses (family *Flaviviridae*, genus *Pestivirus*) are single-strand, positive-sense RNA viruses, whose genome, 12.3 Kb in size, encodes for a polyprotein that is cleaved by viral and cellular proteases in structural (C, E^rns^, E1, E2) and nonstructural (N^pro^, NS2-3, NS4A, NS4B, NS5A, NS5B) proteins (4). Based on the current classification of the International Committee on Taxonomy of Viruses (https://talk.ictvonline.org/taxonomy/), genus *Pestivirus* is composed of 11 recognized species, namely *Pestivirus A* (original designation *Bovine viral diarrhea virus 1*, BVDV-1), *Pestivirus B* (*Bovine viral diarrhea virus 2*, BVDV-2), *Pestivirus C* (*Classical swine fever virus*, CSFV), *Pestivirus D* (*Border disease virus*, BDV), *Pestivirus E* (pronghorn pestivirus), *Pestivirus F* (Bungowannah virus), *Pestivirus G* (giraffe pestivirus), *Pestivirus H* (Hobi-like pestivirus, HoBiPeV), *Pestivirus I* (Aydin-like pestivirus), *Pestivirus J* (rat pestivirus) and *Pestivirus K* (atypical porcine pestivirus) (4). At least three pestivirus species are infecting primarily cattle. BVDV-1 and BVDV-2, known for decades, are grouped in 21 and 3 subtypes, respectively (3, 5, 6). HoBiPeV was first isolated from a contaminated batch of fetal bovine serum (FBS) from Brazil (7) and later detected in a contaminated cell culture and the blood of a buffalo in South America (8). Further HoBiPeV infections were reported in Thailand, Italy, Bangladesh, India, Argentina, China, and Turkey (9–20). However, while viruses circulating in South America, Europe, Thailand and China are closely related, other Asian HoBiPeV strains are highly divergent and at least four different subtypes have been identified so far (3). HoBiPeV infection has the same outcomes reported for BVDV-1 and BVDV-2, including immunosuppression, respiratory disease, gastroenteritis, persistent infections, mucosal disease, and reproductive disorders (10, 15, 19–30). The most relevant economic losses associated with BVDV-1 and BVDV-2 infection are due to reproductive disorders. The infection of pregnant cows during the first trimester of gestation with noncytopathic (ncp) BVDV strains may lead to failure of fertilization, return to oestrus, abortion, congenital malformations, stillbirths, or the birth of persistently infected (PI) animals. PI calves are BVDV seronegative but virus-positive animals, which shed BVDV with their secretion lifelong. They may develop a severe, fatal form of the disease, referred to as mucosal disease, due to superinfection with a cytopathogenic (cp) strain (2, 3). While a plethora of reports are available about reproductive failures caused by pestiviruses belonging to either BVDV-1 or BVDV-2 species, little is presently know about the impact of HoBiPeV infection on cattle reproduction. Therefore, in this paper I provide an up-to-date literature review about the association of this emerging pestivirus to reproductive disorders in cattle.

## Reproductive Disorders After HoBiPeV Natural Infections

The first report of HoBiPeV sequences detected in bovine fetuses dates back to 2006, when a study was published aiming to investigate the genetic variability of BVDV strains from archival samples (31). Two fetuses, aborted in 2002 and 2004, respectively, were later found to contain HoBiPeV strains, which clustered with the virus prototype D32/00_HoBi (7). The fetuses were from farms located in the State of São Paulo, about 100 km apart from each other, suggesting that the two strains were probably circulating among cattle and causing reproductive disorders in the region. However, no further details were reported about the herds of origin of the fetuses.

Reproduction failures associated with natural infection with HoBiPeV were later reported in Italy in 2011 (20). An abortion storm occurred in a cattle herd of southern Italy, which included about 600 Holstein cattle, involving eight multiparous cows at 4–6 months of pregnancy out of 98 pregnant animals. Affected cows did not display any relevant clinical signs before nor any sequelae after the abortion. Two aborted fetuses were submitted to laboratory investigations, displaying high titres of HoBiPeV RNA in their tissues, ranging from 4.31 × 10^2^ (kidney of one fetus) to 5.78 × 10^4^ (lung of the other fetus) RNA copies per μl of template, by a specific real-time RT-PCR assay. No additional pathogens were detected by molecular methods for abortogenic agents of cattle. By immunofluorescence assay using an NS3 monoclonal antibody, a cytoplasmic fluorescence was evident in cryostat sections obtained from the lungs and spleen of the fetuses, whereas successful isolation of a HoBiPeV ncp strain was obtained from the same fetuses. Sequence and phylogenetic analyses of pestivirus's most informative genomic regions (E2, 5'UTR, N^pro^) revealed that the abortogenic HoBiPeV strain was closely related to the virus which had been responsible for the respiratory outbreak a few months before (10).

More data are available about the generation of HoBiPeV PI calves under natural conditions. Pestiviruses can induce immune tolerance in calves born to cows infected in the first 3–4 months of pregnancy. These immune tolerant calves are PI and may have congenital malformations or appear clinically normal and show immunosuppression, thus developing respiratory and gastroenteric disease often caused by opportunistic pathogens (1–3). PI animals have been frequently reported due to BVDV-1, BVDV-2, BDV, CSFV, and, only recently, HoBiPeV infections (22). A calf born in October 2011 in a cattle herd with active HoBiPeV circulation in southern Italy displayed a low birth weight and a decreased growth rate, and later developed respiratory disease with cough and nasal discharge and ruffled hair with alopecic areas in different parts of the body caused by cutaneous mycosis. Laboratory investigations revealed that the calf was pestivirus antibody negative and virus positive, with the strain being characterized as ncp HoBiPeV. In March 2013, at the age of 17 months, the PI calf developed severe clinical signs and subsequently died. A ncp/cp HoBiPeV pair was isolated from the dead animal, which was highly suggestive of mucosal disease (23).

Subsequent reports of mucosal disease in calves in Brazil account for the generation of PI animals as a consequence of congenital infection with HoBiPeV (25, 29), analogous to what was reported for BVDV-1 and BVDV-2 (3). The identification and elimination of PI animals is the main direct prophylactic measure to achieve the eradication of pestivirus infections in cattle herds (32). This strategy has been proven to be useful also against HoBiPeV. In a cattle herd with large economic losses due to reproductive disorders caused by HoBiPeV, the application of an intensive eradication program, based on the detection and slaughtering of PI animals, resulted in a marked improvement of the productive performances (26).

## Reproductive Disorders After HoBiPeV Experimental Infections

Several experimental infections have been carried out to better understand the impact of HoBiPeV infection on cattle reproduction.

In one of these studies, eight pregnant heifers were infected at around day 70 of gestation by intranasal instillation of either Brazilian HoBi_D32/00 or Italian Italy-1/10-1 HoBiPeV isolate (24). The experimental infection was successful in all animals but one inoculated with the Brazilian strain. Another heifer inoculated with HoBi_D32/00 aborted at 8 months of pregnancy, and the fetus was positive for HoBiPeV by RT-PCR. Of the remaining six animals, two heifers infected with the Italian strain gave birth to weak calves infected by HoBiPeV and died within 36 h of birth. In comparison, calves born to the other four animals (two inoculated with the Brazilian strain and two inoculated with the Italian strain) were diagnosed as PI animals by consecutive positive results throughout the study. These PI calves were later housed with pestivirus seronegative calves, sheep, goats, and pigs, resulting in successful infection of all exposed animals (33). Analogous to BVDV-1 and BVDV-2 PI animals, HoBiPeV PI calves displayed extensive depletion in the thymus, with PI calves surviving <5 weeks having lower corticomedullary ratios and greater depletion (34).

In a subsequent study by the same research group (35), cows that had previously calved BVDV-1 (four cows) or BVDV-2 (two cows) PI calves and had high antibody titres against the homologous BVDV strains, were inoculated with HoBiPeV at 85 days of gestation. At 30 days after the challenge, one inoculated cow had no fetus, while the fetuses harvested from 5 of the exposed dams (three BVDV-1 and two BVDV-2 cows) were all positive for HoBiPeV RNA. Therefore, there was no fetal cross-protection elicited by the previous infection with BVDV-1 or BVDV-2 against this emerging group of viruses.

A comprehensive study was carried out in pregnant sheep to evaluate their suitability as a model for vaccination trials of HoBiPeV experimental formulation for cattle (36). In fact, the HoBiPeV challenge of cattle to evaluate the fetal protection induced by pestivirus vaccines require great efforts due to handling a high number of large-size animals, adopting adequate biosecurity measures and spending much money for purchasing and feeding cattle. In the sheep study, ten ewes at different pregnancy stages (30 or 50 days) were experimentally infected with the Italian HoBiPeV prototype isolate Italy-1/10-1. All but one infected ewes underwent reproductive disorders, including abortion (*n* = 3), stillbirth (*n* = 4) or generation of PI lambs (*n* = 4). Aborted fetuses, stillborn and dead lambs displayed extensive histopathological changes, including hemorrhages, congestion and mononuclear infiltration in internal organs, and immunohistochemical detection of pestiviral antigens in affected tissues. PI lambs were constantly viremic, shed the virus through the nasal secretions and feces and, with one exception, were HoBiPeV seronegative. The single seropositive infected lamb showed low-titer viremia and viral shedding that ceased only several weeks after the 3-month observation period. Therefore, the sheep model reproduced the reproductive disorders observed in cattle due to natural or experimental infection with HoBiPeV and represents a suitable model for the evaluation of the fetal protection induced by pestivirus vaccines.

## *In-vitro* Studies Involving the Bovine Reproductive Tract

A study was carried out to evaluate the growth of different bovine pestivirus species in testicle cell cultures obtained from taurine (*Bos taurus taurus*), indicine (*Bos taurus indicus*), and mixed taurine and indicine (*Bos taurus taurus* × *Bos taurus indicus*) breeds (37). In this experiment, the HoBiPeV isolate Italy 1/10-1 was found to replicate efficiently in primary bovine testicle cells, and no significant differences were observed based on breed, whereas, there were significant differences in growth rate among animals within each breed group.

Another study aimed to evaluate the effect of pestivirus infection on embryo development as a consequence of exposure of bovine oocytes to different pestivirus species (38). Bovine oocytes were collected after slaughtering and treated with different concentrations of pestiviruses (BVDV-1, BVDV-2 and HoBiPeV) during their *in-vitro* maturation. After *in-vitro* fertilization, zygotes were cultured for seven days, and the developed embryos were evaluated according to the guidelines of the International Embryo Transfer Society and submitted to pestivirus detection by real-time RT-PCR. The obtained results showed that *in-vitro* produced embryos from BVDV-1 and BVDV-2 infected oocytes developed normally, but were infected, thus carrying the virus. In contrast, HoBiPeV infected oocytes had reduced cleavage and cause pre-implantation embryo loss, but viable embryos did not carry the virus, suggesting that HoBiPeV infection may cause embryo loss before blastocyst development. Therefore, the study demonstrated that oocyte exposure to HoBiPeV resulted in reduced embryo development, having the potential to impact more severely on cattle reproduction than other bovine pestiviruses.

## Discussion

Pestivirus infections cause economically significant diseases affecting the cattle industry, with losses stemming from decreased production and reproductive performance, and control costs. Economic losses associated with reproduction are relevant since these viruses may have negative effects on all phases of bovine reproduction. Reduced conception rates, early embryonic death, abortion, congenital defects, and weak calves have all been associated with pestivirus infection of susceptible cows. Besides, the birth of PI calves as a consequence of *in utero* fetal exposure is critical in the perpetuation of the virus in an infected herd or spread to other susceptible herds (39, 40). All studies reporting the outcomes of naturally or experimentally occurring HoBiPeV infection in cattle account for a high pathogenic potential of this virus on the reproduction, with the development of the same reproductive disorders that are commonly observed during BVDV-1 and BVDV-2 infection (20, 22, 26, 35). In addition, the virus affected embryo development as a result of oocyte exposure.

Consequently, this group of emerging viruses should be considered among bovine pathogens that impact reproduction and be included in the diagnostic algorithms of reproductive disorders in cattle. A major issue in HoBiPeV detection is that antigenic, molecular, and serological tests commercially available for BVDV-1 and BVDV-2 may not react or react at a lower extent with this emerging virus (24, 41). Also, even assays specifically developed for HoBiPeV detection may fail to detect the more divergent strains identified in recent times (42). Therefore, there is a need in the improvement of HoBiPeV specific diagnostic assays in order not to misdiagnose the reproductive disorder caused by this virus, especially in areas with low virus circulation. Nevertheless, vaccination trials are also required to assess the efficacy of BVDV currently available vaccines against HoBiPeV infections, since there are some concerns that those vaccines may be not completely protective against this pestivirus (35, 43).

## Author Contributions

ND conceived and wrote the article.

## Conflict of Interest

The author declares that the research was conducted in the absence of any commercial or financial relationships that could be construed as a potential conflict of interest.

## References

[1] Ridpath J. The contribution of infections with bovine viral diarrhea viruses to bovine respiratory disease. Vet Clin North Am Food Anim Pract. (2010) 26:335–48. 10.1016/j.cvfa.2010.04.003 20619188

[2] Brock KV. The many faces of bovine viral diarrhea virus. Vet Clin North Am Food Anim Pract. (2004) 20:1–3. 10.1016/j.cvfa.2003.12.002 15062470

[3] Evans CA Pinior B Larska M Graham D Schweizer M Guidarini C. Global knowledge gaps in the prevention and control of bovine viral diarrhoea (BVD) virus. Transbound Emerg Dis. (2019) 66:640–52. 10.1111/tbed.13068 30415496

[4] Smith DB Meyers G Bukh J Gould EA Monath T Scott Muerhoff A. Proposed revision to the taxonomy of the genus *Pestivirus*, family *Flaviviridae*. J Gen Virol. (2017) 98:2106–12. 10.1099/jgv.0.000873 28786787 PMC5656787

[5] Lanave G Decaro N Lucente MS Guercio A Cavaliere N Purpari G. Circulation of multiple subtypes of bovine viral diarrhoea virus type 1 with no evidence for HoBi-like pestivirus in cattle herds of southern Italy. Infect Genet Evol. (2017) 50:1–6. 10.1016/j.meegid.2017.02.009 28189886

[6] Decaro N Lucente MS Lanave G Gargano P Larocca V Losurdo M. Evidence for circulation of bovine viral diarrhoea virus type 2c in ruminants in southern Italy. Transbound Emerg Dis. (2017) 64:1935–44. 10.1111/tbed.12592 27878974

[7] Schirrmeier H Strebelow G Depner K Hoffmann B Beer M. Genetic and antigenic characterization of an atypical pestivirus isolate, a putative member of a novel pestivirus species. J Gen Virol. (2004) 85:3647–52. 10.1099/vir.0.80238-0 15557237

[8] Stalder H Meier P Pfaffen G Wageck-Canal C Rüfenacht J Schaller P. Genetic heterogeneity of pestiviruses of ruminants in Switzerland. Prev Vet Med. (2005) 72:37–41. 10.1016/j.prevetmed.2005.01.020 16213615

[9] Liu L Kampa J Belák S Baule C. Virus recovery and full-length sequence analysis of atypical bovine pestivirus Th/04_KhonKaen. Vet Microbiol. (2009) 138:62–8. 10.1016/j.vetmic.2009.03.006 19349128

[10] Decaro N Lucente MS Mari V Cirone F Cordioli P Camero M. Atypical pestivirus and severe respiratory disease in calves, Europe. Emerg Infect Dis. (2011) 17:1549–52. 21801648 10.3201/eid1708.101447 PMC3381567

[11] Decaro N Mari V Lucente MS Sciarretta R Elia G Ridpath JF. Detection of a Hobi-like virus in archival samples suggests circulation of this emerging pestivirus species in Europe prior to 2007. Vet Microbiol. (2013) 167:307–13. 10.1016/j.vetmic.2013.09.006 24095625

[12] Mishra N Rajukumar K Pateriya A Kumar M Dubey P Behera SP. Identification and molecular characterization of novel and divergent HoBi-like pestiviruses from naturally infected cattle in India. Vet Microbiol. (2014) 174:239–46. 10.1016/j.vetmic.2014.09.017 25301283

[13] Haider N Rahman MS Khan SU Mikolon A Gurley ES Osmani MG. Identification and epidemiology of a rare HoBi- like pestivirus strain in Bangladesh. Transbound Emerg Dis. (2014) 6:193–8. 10.1111/tbed.12218 24650238

[14] Shi H Kan Y Yao L Leng C Tang Q Ji J. Identification of natural infections in sheep/goats with HoBi-like pestiviruses in China. Transbound Emerg Dis. (2016) 63:480–4. 10.1111/tbed.12551 27478131

[15] Hoppe IBAL Souza-Pollo A Medeiros ASR Samara SI Carvalho AAB. HoBi-like pestivirus infection in an outbreak of bovine respiratory disease. Res Vet Sci. (2019) 126:184–91. 10.1016/j.rvsc.2019.09.003 31539795

[16] Shi H Li H Zhang Y Yang L Hu Y Wang Z. Genetic diversity of bovine pestiviruses detected in backyard cattle farms between 2014 and 2019 in Henan Province, China. Front Vet Sci. (2020) 7:197. 10.3389/fvets.2020.00197 32363203 PMC7181229

[17] Pecora A Perez Aguirreburualde MS Ridpath JF Dus Santos MJ. Molecular characterization of pestiviruses in fetal bovine sera originating from Argentina: evidence of circulation of HoBi-Like viruses. Front Vet Sci. (2019) 6:359. 10.3389/fvets.2019.00359 31681812 PMC6805694

[18] Timurkan MÖ Aydin H. Increased genetic diversity of BVDV strains circulating in Eastern Anatolia, Turkey: first detection of BVDV-3 in Turkey. Trop Anim Health Prod. (2019) 51:1953–61. 10.1007/s11250-019-01901-6 31055737

[19] Chen M Liu M Liu S Shang Y. HoBi-like pestivirus infection leads to bovine death and severe respiratory disease in China. Transbound Emerg Dis. (2020). 10.1111/tbed.13832. [Epub ahead of print]. 32926568

[20] Decaro N Lucente MS Mari V Sciarretta R Pinto P Buonavoglia D. Hobi-like pestivirus in aborted bovine fetuses. J Clin Microbiol. (2012) 50:509–12. 10.1128/JCM.05887-11 22162547 PMC3264192

[21] Decaro N Mari V Pinto P Lucente MS Sciarretta R Cirone F. Hobi-like pestivirus: both biotypes isolated from a diseased animal. J Gen Virol. (2012) 93:1976–83. 10.1099/vir.0.044552-0 22764319

[22] Decaro N Losurdo M Lucente MS Sciarretta R Mari V Larocca V. Persistent infection caused by Hobi-like pestivirus. J Clin Microbiol. (2013) 51:1241–3. 10.1128/JCM.03134-12 23325822 PMC3666814

[23] Decaro N Lanave G Lucente MS Mari V Varello K Losurdo M. Mucosal disease-like syndrome in a calf persistently infected by Hobi-like pestivirus. J Clin Microbiol. (2014) 52:2946–54. 10.1128/JCM.00986-14 24899039 PMC4136150

[24] Bauermann FV Falkenberg SM Vander Ley B Decaro N Brodersen BW Harmon A. Generation of calves persistently infected with HoBi-like pestivirus and comparison of methods for detection of these persistent infections. J Clin Microbiol. (2014) 52:3845–52. 10.1128/JCM.01563-14 25122860 PMC4313213

[25] Weber MN Mósena AC Simões SV Almeida LL Pessoa CR Budaszewski RF. Clinical presentation resembling mucosal disease associated with ‘HoBi'-like pestivirus in a field outbreak. Transbound Emerg Dis. (2016) 63:92–100. 10.1111/tbed.12223 24735072

[26] Decaro N Lucente MS Losurdo M Larocca V Elia G Occhiogrosso L. HoBi-Like pestivirus and its impact on cattle productivity. Transbound Emerg Dis. (2016) 63:469–73. 10.1111/tbed.12529 27390140

[27] Cortez A Araújo JP Jr Flores EF Ribeiro MG Megid J Paes AC. Complete genome sequence of a Hobi-like virus isolated from a nelore cow with gastroenteric disease in the State of São Paulo, Brazil. Genome Announc. (2017) 5:e00767–17. 10.1128/genomeA.00767-17 28818893 PMC5604766

[28] Jardim JC Amaral BP Martins M Sebastian P Heinemann MB Cortez A. Respiratory signs, fever and lymphopenia in calves inoculated with Brazilian HoBi-like pestiviruses. Microb Pathog. (2018) 123:264–8. 10.1016/j.micpath.2018.07.024 30040999

[29] Cruz RAS Rodrigues WB Silveira S Oliveira VHS Campos CG Leite Filho RV. Mucosal disease-like lesions caused by HoBi-like pestivirus in Brazilian calves in 2010-2011: clinical, pathological, immunohistochemical, and virological characterization. Res Vet Sci. (2018) 119:116–21. 10.1016/j.rvsc.2018.06.010 29913325

[30] Weber MN Bauermann FV Canal CW Bayles DO Neill JD Ridpath JF. Temporal dynamics of 'HoBi'-like pestivirus quasispecies in persistently infected calves generated under experimental conditions. Virus Res. (2017) 227:23–33. 10.1016/j.virusres.2016.09.018 27693289

[31] Cortez A Heinemann MB De Castro MG Soares RM Pinto AM Alfieri AA Genetic characterization of Brazilian bovine viral diarrhea virus isolates by partial nucleotide sequencing of the 5'-UTR region. Pesq Vet Bras. (2006) 26:211–6. 10.1590/S0100-736X2006000400005

[32] Bauermann FV Ridpath JF Weiblen R Flores EF. HoBi-like viruses: an emerging group of pestiviruses. J Vet Diagn Invest. (2013) 25:6–15. 10.1177/1040638712473103 23345268

[33] Bauermann FV Falkenberg SM Decaro N Flores EF Ridpath JF Experimental infection of calves, sheep, goats and pigs with HoBi-like viruses by direct inoculation or exposure to persistently infected calves. Vet Microbiol. (2015) 181:289–93. 10.1016/j.vetmic.2015.10.011 26525738

[34] Falkenberg SM Bauermann FV Ridpath JF Characterization of thymus-associated lymphoid depletion in bovine calves acutely or persistently infected with bovine viral diarrhea virus 1, bovine viral diarrhea virus 2 or HoBi-like pestivirus. Arch Virol. (2017) 162:3473–80. 10.1007/s00705-017-3523-x 28795249 PMC5640739

[35] Bauermann FV Falkenberg SM Ridpath JF. HoBi-like virus RNA detected in foetuses following challenge of pregnant cows that had previously given birth to calves persistently infected with bovine viral diarrhoea virus. Transbound Emerg Dis. (2017) 64:1624–32. 10.1111/tbed.12556 27615437

[36] Decaro N Losurdo M Larocca V Lucente MS Mari V Varello K. HoBi-like pestivirus experimental infection in pregnant ewes: reproductive disorders and generation of persistently infected lambs. Vet Microbiol. (2015) 178:173–80. 10.1016/j.vetmic.2015.05.011 26013415 PMC7172706

[37] Weber MN Bauermann FV Gómez-Romero N Herring AD Canal CW Neill JD. Variation in pestivirus growth in testicle primary cell culture is more dependent on the individual cell donor than cattle breed. Vet Res Commun. (2017) 41:1–7. 10.1007/s11259-016-9666-5 27864728

[38] da Silva Cardoso Pinto V Alves MF de Souza Nunes Martins M Basso AC Tannura JH Pontes JHF. Effects of oocytes exposure to bovine diarrhea viruses BVDV-1, BVDV-2 and Hobi-like virus on *in vitro*-produced bovine embryo development and viral infection. Theriogenology. (2017) 97:67–72. 10.1016/j.theriogenology.2017.04.028 28583610

[39] Grooms DL. Reproductive consequences of infection with bovine viral diarrhea virus. Vet Clin North Am Food Anim Pract. (2004) 20:5–19. 10.1016/j.cvfa.2003.11.006 15062471

[40] Newcomer BW Walz PH Givens MD Wilson AE Efficacy of bovine viral diarrhea virus vaccination to prevent reproductive disease: a meta-analysis. Theriogenology. (2015) 83:360–5.e1. 10.1016/j.theriogenology.2014.09.028 25447148

[41] Moorthy D Mishra N Kalaiyarasu S Jhade SK Singh VP. Evaluation of currently available bovine viral diarrhoea virus (BVDV) and HoBi-like pestivirus (HoBiPeV) specific diagnostic tests in detection of highly divergent HoBiPeVs in cattle. J Virol Methods. (2019) 272:113707. 10.1016/j.jviromet.2019.113707 31351170

[42] Losurdo M Mari V Lucente MS Colaianni ML Padalino I Cavaliere N. Development of a TaqMan assay for sensitive detection of all pestiviruses infecting cattle, including the emerging HoBi-like strains. J Virol Methods. (2015) 224:77–82. 10.1016/j.jviromet.2015.08.013 26300370 PMC7113749

[43] Decaro N Mari V Sciarretta R Lucente MS Camero M Losurdo M. Comparison of the cross-antibody response induced in sheep by inactivated bovine viral diarrhoea virus 1 and Hobi-like pestivirus. Res Vet Sci. (2013) 94:806–8. 10.1016/j.rvsc.2012.11.016 23261155

